# Career Anxiety and Career Decision-Making Difficulties Among Female Undergraduates in Four Non-Elite Chinese Universities: A Mixed-Methods Study

**DOI:** 10.3390/bs16091479

**Published:** 2026-08-25

**Authors:** Jiadan Pan, Mu He

**Affiliations:** 1 Faculty of Education, Tianjin Normal University, Tianjin 300387, China; panjiadan@stu.tjnu.edu.cn; 2 School of Education Science, Huaiyin Normal University, Huai’an 223300, China

**Keywords:** career anxiety, career decision-making difficulties, career decision-making self-efficacy, parental career support, female undergraduates in non-elite universities

## Abstract

Career decision-making difficulties (CDDs) among female undergraduates are associated with both individual psychological processes and culturally embedded social contexts. This study examined the associations among career anxiety, career decision-making self-efficacy (CDSE), perceived frequency of parental career support, and CDD among female undergraduates from four purposively selected non-elite universities in Jiangsu Province, China. Guided by Social Cognitive Career Theory (SCCT), this explanatory sequential mixed-methods study combined quantitative and qualitative approaches. Quantitative data from 407 participants were analyzed using SPSS 27.0, AMOS 26.0, and PROCESS macro 4.1, including confirmatory factor analysis and conditional process analysis with 5000 bootstrap samples, followed by thematic analysis of semi-structured interviews with 12 participants using NVivo 12. Career anxiety was positively associated with CDD and negatively associated with CDSE, with CDSE partially accounting for the association between anxiety and decision difficulties. The anxiety–CDD association was stronger among students reporting higher frequencies of parental career support behaviors. Qualitative findings suggested that perceived support quality, autonomy support, and contextual fit shaped how parental involvement was experienced. These findings suggest that parental career support should be understood not only in terms of behavioral frequency but also in relation to how involvement is experienced under conditions of heightened career anxiety. Longitudinal, dyadic, and culturally diverse research is needed.

## 1. Introduction

The rapid expansion of higher education in China has substantially increased the number of university graduates, with official statistics indicating continued growth in higher education enrollment and graduate output (Ministry of Education of the People’s Republic of China, 2025). This expansion has intensified competition in the graduate labor market and has contributed to structural imbalances between graduate supply and demand. Importantly, however, this expansion has not produced a homogeneous graduate population. China’s higher education system is organized around a steep institutional hierarchy, and this hierarchy is closely associated with labor-market opportunities. Among college graduates seeking state-sector employment, those from elite 211-designated universities were more likely to receive job offers than their non-elite counterparts—61% versus 46%—suggesting that institutional prestige remains an important screening criterion in graduate recruitment (Li et al., 2024). For female graduates at non-elite institutions, these institutional disadvantages may intersect with gendered labor-market constraints, as school tier has been shown to moderate the gender gap in access to institutional employment, with the gap widening among graduates from non-Double First-Class universities (Zhu & Li, 2025). It is within this doubly stratified context—institutional hierarchy intersecting with gender inequality—that the present study examines career decision-making difficulties among female undergraduates sampled from four purposively selected non-elite universities in Jiangsu Province, China.

Within this context, female undergraduates at non-elite universities may experience a form of “double disadvantage,” referring here to the intersection of gender inequality and institutional stratification. In this study, the term describes the structural context defined by the sampling design rather than an individual-level explanatory construct.

Within Chinese collectivistic family norms, filial expectations and partial economic dependence on parents during undergraduate years may render female undergraduates from local non-elite universities particularly sensitive to parental career-related expectations, as such expectations may be experienced not only as support but also as implicit obligations to conform to family-preferred career pathways (e.g., stable employment, geographic proximity, or state-sector positions). Parental career involvement, even when well-intentioned, may thus carry relational obligations that shape how students experience career-related pressure under labor-market disadvantage.

This contextual condition defines both the population of interest and the scope of empirical interpretation. Rather than being modeled statistically, it serves as the structural background within which the associations among career anxiety, career decision-making self-efficacy, parental career support, and career decision-making difficulties are examined.

Career anxiety has been widely discussed as an affective factor associated with career decision-making difficulties (CDD). Previous research suggests that anxiety is related to avoidance tendencies and reduced decision-making confidence, both of which may be associated with greater difficulty in career decision-making (Anderson, 2003; Saka & Gati, 2007). To examine these relationships, this study draws on Social Cognitive Career Theory (SCCT; Lent et al., 1994), which provides a useful framework for understanding how affective experiences and self-efficacy beliefs are associated with career decision-making processes. In particular, career decision-making self-efficacy (CDSE) is considered a potential psychological pathway through which career anxiety may be indirectly associated with CDD.

In addition, this study examines the perceived frequency of parental career support as a possible boundary condition in the association between career anxiety and CDD. Within the high-involvement Chinese family context, parental career support is often viewed as a facilitative resource. For many female undergraduates in non-elite local universities, however, career decisions may be embedded in intergenerational expectations, economic dependence during university, and culturally valued obligations to respond to parental concern. Parents’ career-related involvement may therefore be interpreted not only as informational or emotional support, but also as a relational expectation concerning stability, geographic proximity, or state-sector employment. This socio-cultural positioning may make students more sensitive to parental advice, especially when they already perceive themselves as disadvantaged in the graduate labor market. Therefore, the present study distinguishes the frequency or extent of perceived parental career support from the quality, autonomy-supportiveness, or helpfulness of that support. Rather than assuming a uniformly protective role, this study examines whether the direct association between career anxiety and CDD varies according to perceived parental career support frequency.

Guided by SCCT and situated within the structural context of double disadvantage, this study addresses the following research questions:

RQ1: Is career anxiety indirectly associated with CDD through CDSE among female undergraduates sampled from four purposively selected non-elite universities in Jiangsu Province, China?

RQ2: Does the perceived frequency of parental career support moderate the direct association between career anxiety and CDD?

RQ3: How do female undergraduates with high career anxiety subjectively perceive and interpret the role of parental career support in their career decision-making processes?

Building on these questions, this study specifies a conditional process model containing an unconditional indirect association through CDSE and a moderated direct association between career anxiety and CDD. The quantitative phase examines the indirect association involving CDSE and whether the direct career anxiety–CDD association differs by levels of perceived parental career support frequency. The qualitative phase uses semi-structured interviews to elaborate participants’ plausible subjective interpretations of the observed quantitative pattern. These qualitative themes are not treated as evidence of mediating mechanisms or population-level explanations, but as contextualized accounts of how some participants made sense of parental career support in relation to their career decision-making experiences.

## 2. Literature Review

### 2.1. Theoretical Framework: SCCT and Structural Context

This study draws on Social Cognitive Career Theory (SCCT; Lent et al., 1994) to frame the psychological mechanisms associated with CDD among female undergraduates in non-elite Chinese universities. SCCT conceptualizes career behavior as shaped by interactions among contextual conditions, cognitive mechanisms, and behavioral outcomes. Although early SCCT formulations emphasized self-efficacy, outcome expectations, and goals, subsequent work explicitly extended the framework to contextual supports and barriers (Lent et al., 2000). Later developments—especially the Career Self-Management model (Lent & Brown, 2013) and Lent’s (2020) integrative overview—further strengthened the theory’s treatment of contextual barriers, subjective appraisals, and adaptive coping.

SCCT is particularly well-suited to the present study because it explicitly theorizes how structural disadvantages may be associated with the career development process. Within SCCT, contextual barriers may be related to career behavior either directly or indirectly through person-cognitive variables such as CDSE. For female undergraduates at non-elite institutions, the intersection of institutional hierarchy and gender inequality can be understood as a design-level structural context in which career-related anxiety, self-efficacy beliefs, and decisional difficulties are examined. The present study examines both pathways: a direct association of career anxiety with CDD, and an indirect association through CDSE. It further examines whether a key contextual resource—parental career support—moderates the strength of the direct path, consistent with SCCT’s proposition that social supports may condition the impact of barriers on career behavior (Lent et al., 2000; Lent & Brown, 2013). In the present study, “double disadvantage” is treated as a design-level contextual condition rather than an explanatory variable: it refers to the intersection of institutional stratification and gender inequality and defines the population within which focal psychological mechanisms are examined.

### 2.2. Career Anxiety and Career Decision-Making Difficulties

Career anxiety refers to negative emotions arising in the course of career decision-making (Shin & Lee, 2019), including fear regarding one’s future career (Vignoli et al., 2005). In China’s massified higher education system, career anxiety may be intensified by institutional pressure, labor-market competition, and concerns about future security.

In this study, the association between career anxiety and CDD is understood through two complementary theoretical explanations. First, career anxiety may be associated with hesitation, avoidance, and emotional disruption in the decision-making process (Saka & Gati, 2007). Recent work on career indecision in high-school and vocational-training contexts also indicates continuing scholarly attention to anxiety-related and self-evaluative difficulties during educational-to-career transitions (Mata-Correas et al., 2025). Second, anxiety may impair attentional control and working memory, thereby weakening the cognitive resources required for evaluating career options (Eysenck & Calvo, 1992; Eysenck et al., 2007). Prior studies also show that elevated anxiety is associated with lower career certainty (Fuqua et al., 1987; Campagna & Curtis, 2007). Taken together, this literature suggests that career anxiety is an important affective factor associated with CDD.

CDD, the focal outcome of this study, is conceptualized using Gati et al.’s (1996) taxonomy and measured with the Chinese Career Decision-Making Difficulties Questionnaire (Wu et al., 2016).

### 2.3. Career Decision-Making Self-Efficacy as a Mediator

Within SCCT, affective states such as anxiety are a major source of self-efficacy beliefs (Lent et al., 1994). Career decision-making self-efficacy (CDSE), defined as confidence in one’s ability to complete career-related tasks (Taylor & Betz, 1983), therefore provides a plausible cognitive mechanism linking anxiety to decisional outcomes. In this study, CDSE is measured using Peng and Long’s (2001) localized measure. Previous research consistently links higher CDSE to lower CDD and greater decisional readiness (Guay et al., 2006; Choi et al., 2012; Jaensch et al., 2015; Kim et al., 2014). More recent meta-analytic evidence further supports this link: Udayar et al. (2020) found that process-related self-efficacy showed stronger negative associations with career indecision than generalized self-efficacy, content-related self-efficacy, or self-esteem.

At the same time, the mediating role of CDSE may be insufficient under conditions of heightened contextual pressure. The Career Self-Management model (Lent & Brown, 2013) suggests that strong contextual barriers may disrupt adaptive career behavior in ways that are not fully explained by self-efficacy alone. Career anxiety may therefore be associated with lower CDSE while also remaining directly associated with CDD. Testing this partial mediation pattern is a central aim of the present study.

### 2.4. Parental Career Support as a Conditional Resource

Parental career support has been conceptualized in multiple ways. Turner et al. (2003), drawing on Bandura’s (1997) self-efficacy theory, identified four dimensions: instrumental assistance, career-related modeling, verbal encouragement, and emotional support. Leung and Zhou (2025) proposed a complementary framework comprising action support, autonomy-affirming support, and emotional support. Across SCCT-related research, parental career support is generally regarded as a facilitating resource associated with stronger CDSE and lower CDD (Z. Liu & Liang, 2025; A. Zhou et al., 2024).

However, parental support may not operate uniformly across contexts. Dietrich and Kracke (2009) conceptualized parental support and parental interference as independent but potentially co-occurring dimensions. They further distinguished parental support from parental interference and lack of engagement, showing that support was positively associated with career exploration, whereas interference and lack of engagement were associated with decision-making difficulties. This distinction is especially relevant in contexts of high parental involvement. In contemporary Chinese families, supportive behaviors may coexist with directive guidance or implicit pressure. Consistent with this view, Parola and Marcionetti (2022) found that the developmental value of parental involvement depends on its form rather than its mere presence, while X. Tian et al. (2021) showed that parenting styles influence career decision-making difficulties through core self-evaluation and career calling. For students experiencing elevated career anxiety, parental involvement may be less protective, particularly when it is perceived as poorly matched to their needs, autonomy-constraining, or psychologically burdensome.

Accordingly, this study adopts a support-context fit perspective, consistent with Self-Determination Theory’s distinction between autonomy-supportive and controlling environments (Ryan & Deci, 2000). The central issue is not simply whether parental career support is present, but under what conditions it functions as a resource rather than an added source of strain.

A further measurement limitation in the existing literature warrants attention. The most widely used parental career support instruments—including the behavioral subscale adopted in the present study (Leung & Zhou, 2025)—capture the frequency of support behaviors rather than their perceived quality, autonomy-supportiveness, or contextual fit. This distinction is theoretically consequential: within Self-Determination Theory (Ryan & Deci, 2000), the functional impact of support depends not on its quantity but on whether it is experienced as autonomy-affirming or controlling. Existing quantitative studies are therefore structurally limited in their ability to distinguish between support that facilitates career agency and support that, under conditions of elevated anxiety, may be experienced as burdensome or mismatched to the student’s needs. The present study acknowledges this constraint and interprets the moderation finding accordingly; qualitative data collected in Phase 2 were used to provide contextual interpretations of how some participants experienced parental involvement, rather than to establish a mediating mechanism.

Although parental career support has generally been conceptualized as a protective contextual resource within SCCT, its effects may depend on how such support is interpreted and experienced by students. In family contexts characterized by strong intergenerational interdependence and parental involvement, supportive behaviors may simultaneously provide informational and emotional resources while also introducing expectations or perceived obligations. Therefore, the present study does not assume that parental career support operates exclusively as a buffering factor. Instead, it examines whether parental career support functions as a contextual moderator that alters the strength of the association between career anxiety and CDD. The direction of this moderation was examined empirically.

### 2.5. Research Gaps and the Present Study

Despite substantial research on career anxiety, CDSE, and CDD, three gaps remain.

First, there is a contextual gap. Existing SCCT-based studies in China have predominantly sampled students from elite or key universities, treating the college student population as institutionally homogeneous (e.g., Guan et al., 2017; X. Liu et al., 2024). As X. Liu et al. (2024) explicitly acknowledged, psychological outcomes such as stress and self-efficacy may differ substantially among students at non-elite institutions, yet this population remains empirically underexamined. Female students at non-elite universities, who may experience intersecting institutional and gender-related constraints, are particularly underrepresented in the career development literature (e.g., L. Tian & Hou, 2023; Wu et al., 2016; Z. Zhou, 2024).

Second, there is a theoretical gap. Although parental career support is usually treated as beneficial, less is known about the conditions under which intensive parental involvement may become burdensome rather than protective.

Third, there is a methodological gap. Most prior studies rely on cross-sectional quantitative designs and are less able to capture culturally embedded subjective experiences or to explain unexpected statistical patterns.

To address these gaps, this study employs an explanatory sequential mixed-methods design and tests a moderated mediation model grounded in SCCT. The following hypotheses are proposed:

**H1.**  *Career anxiety is significantly and positively associated with CDD.*

**H2.**  *CDSE partially accounts for the association between career anxiety and CDD.*

**Figure 1:**
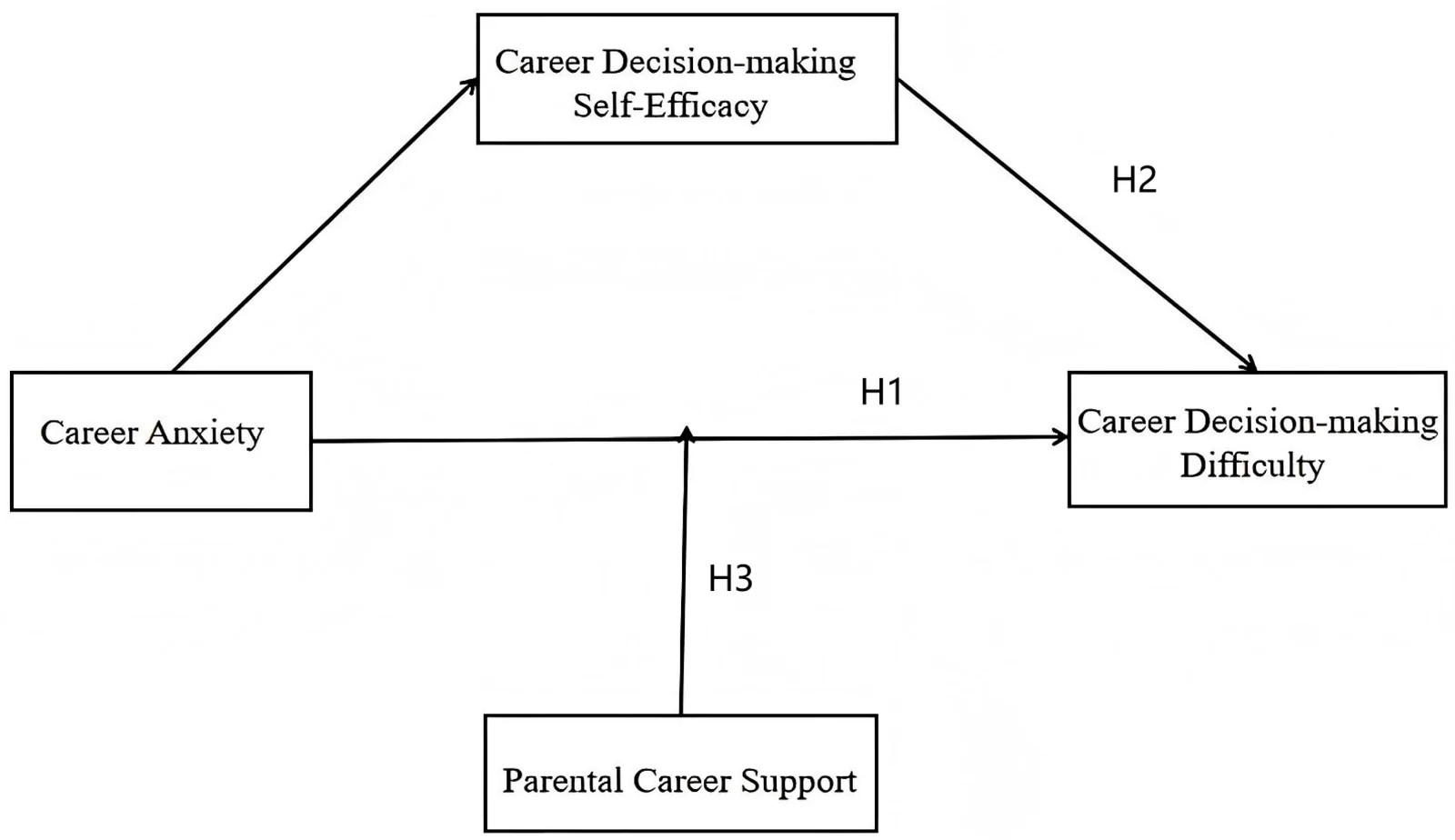
Hypothesized Conditional Process Model. Note. H1 represents the direct association between career anxiety and CDD. H2 represents the indirect association between career anxiety and CDD through CDSE. H3 represents the moderating role of parental career support frequency on the direct career anxiety–CDD association. Control variables are omitted from the figure for clarity.

**H3.**  *The perceived frequency of parental career support significantly moderates the association between career anxiety and CDD, such that the strength of the career anxiety* *–CDD association varies as a function of perceived parental career support frequency (see Figure 1).*

## 3. Materials and Methods

### 3.1. Quantitative Phase

#### 3.1.1. Participants

Given the contextually embedded explanatory design of this study, which seeks to understand career decision-making processes within specific institutional contexts, a purposive maximum variation sampling strategy was employed (Patton, 2015). Specifically, we first defined a bounded target population—female undergraduates enrolled in non-“Double First-Class” local universities—and then constructed a two-dimensional sampling frame based on regional economic gradient and institutional disciplinary attributes. Within this frame, four local undergraduate institutions in Jiangsu Province were purposively selected to capture meaningful variation in labor-market environments and disciplinary cultures while remaining comparable in institutional status (see Table 1). In this way, the sampling strategy captures the study’s focal structural context at the design level. The purpose was not to treat institutional context as an individual-level variable, but to examine the associations among individual-level psychological and social factors within a defined institutional context. 

This sampling logic followed three criteria. First, institutional comparability was maintained by restricting all sampled institutions to non-“Double First-Class” local undergraduate universities with similar regional-service and applied-education missions. This criterion ensured that participants were situated in a different position within China’s hierarchical higher education system, compared with students from “Double First-Class” universities, thereby defining a shared institutional context distinct from research-intensive “Double First-Class” universities. Second, regional variation was introduced by selecting institutions from Southern, Central, and Northern Jiangsu, representing relatively developed, transitional, and less-developed regional environments, respectively. Third, disciplinary variation was incorporated through the inclusion of polytechnic, teacher education, and comprehensive institutions, allowing the study to capture differences in professional culture and gender-related professional expectations and career contexts.

Through this design, the sample does not aim to represent the statistical average of all Chinese universities. Rather, it provides analytically useful contrasts within a bounded population, enabling the study to examine whether the observed associations are consistent across varied regional and disciplinary contexts.

Data collection was conducted between September and October 2025 via the online platform “QuestionStar.” To ensure sample diversity within the selected sites, a counselor-assisted distribution strategy was employed. We liaised with administrative staff responsible for student affairs at the four universities, who disseminated the survey link to female undergraduates across different year levels via administrative notification systems. The study adhered to the Declaration of Helsinki; all participants provided informed consent regarding anonymity, confidentiality, and their right to withdraw.

A total of 476 questionnaires were retrieved. After excluding 69 invalid responses based on the predefined criteria, namely completion time < 180 s or failure on consistency checks, 407 valid questionnaires were retained. This yielded a valid data retention rate of 85.5% among returned questionnaires. Because the survey link was distributed through counselor-assisted notification channels and the total number of students who received or viewed the invitation could not be precisely determined, this percentage should not be interpreted as a response rate. An a priori power analysis (G*Power 3.1) indicated that a minimum sample size of 92 was required for the proposed linear regression models (5 predictors, α = 0.05, power = 0.80, medium effect size f^2^ = 0.15). Our final sample (*N* = 407) substantially exceeds this threshold, ensuring robust statistical power.

Missing data were examined before data cleaning and statistical analysis. Because the online questionnaire was set to require responses to all scale items and demographic questions, no item-level missing data were present among the submitted questionnaires. After excluding invalid responses based on the predefined criteria, all analyses were conducted using the complete valid sample of 407 participants. Therefore, neither listwise deletion due to missing values nor full-information maximum-likelihood estimation was required.

Among participants: 92 were first-year undergraduates (22.60%), 149 were second-year undergraduates (36.61%), 93 were third-year undergraduates (22.85%), and 73 were fourth-year undergraduates (17.94%); 78 humanities students (19.16%), 198 science and engineering students (48.65%), 60 arts and sports students (14.74%), and 71 students from other disciplines (17.44%); 126 only daughters (30.96%), 281 non-only daughters (69.04%).

#### 3.1.2. Measures

Career Anxiety. This study employed the 25-item Career Anxiety Scale developed by Tsai et al. (2017). This instrument assesses four distinct dimensions: (1) personal ability (e.g., “If I possessed professional qualifications, I would not worry about future employment”), (2) irrational beliefs about employment (e.g., “I worry about future employment because I fear new environments”), (3) employment environment (e.g., “I worry about future employment because an economic downturn may occur”), and (4) professional education and training (e.g., “I worry about future employment because I feel uncertain about my professional knowledge and interests”). Items are rated on a 4-point Likert scale (1 = strongly disagree, 4 = strongly agree), with higher aggregate scores reflecting elevated anxiety levels. Tsai et al. (2017) demonstrated acceptable psychometric properties for the scale, reporting Cronbach’s α coefficients between 0.76 and 0.89 across dimensions. The scale’s cross-cultural applicability is further supported by Müceldili et al. (2025), who adopted a revised version featuring a five-item “Employment Environment” subscale (analogous to the employment context dimension) in a Turkish undergraduate sample, reporting acceptable reliability and model fit.

To ensure linguistic equivalence and cultural applicability within the mainland Chinese context, a rigorous translation and back-translation procedure was strictly followed, adhering to the standard guidelines for cross-cultural adaptation of self-report measures (Beaton et al., 2000). First, two bilingual postgraduates majoring in psychology translated the original scale into Chinese independently. A synthesized version was then agreed upon after resolving minor discrepancies. Second, two independent bilingual researchers (blind to the original scale) back-translated the synthesized version into English. Semantic equivalence was verified by comparing the back-translation with the original source. Third, an expert panel—comprising the translators, a psychometrics methodologist, and two experienced career counseling psychologists—reviewed all translated versions alongside the original instrument. This committee systematically evaluated semantic, idiomatic, and conceptual equivalence to resolve any cross-cultural ambiguities, thereby generating the pre-final Chinese version. Finally, rather than a standard pilot test, cognitive interviews (utilizing a think-aloud protocol) were conducted with a purposive sample of 15 female undergraduates from the target demographic. This step ensured that the target population comprehended the items exactly as intended, allowing us to adjust minor phrasing for maximum clarity and verify that the items authentically resonated with the specific linguistic habits of students in the Yangtze River Delta region.

In the present study, the overall Cronbach’s α coefficient for the scale was 0.792, indicating acceptable internal consistency. Regarding construct validity, exploratory factor analysis (EFA) revealed a KMO value of 0.808 and a significant Bartlett’s sphericity test (*p* < 0.001), confirming suitability for factor analysis. Confirmatory factor analysis (CFA) results indicated good model fit (χ^2^/df = 2.405, CFI = 0.967, TLI = 0.954, RMSEA = 0.059, SRMR = 0.030), demonstrating sound reliability and validity.

Career Decision-making Self-efficacy: This study employed the CDSE Scale developed by Peng and Long (2001). Although a localized instrument, its development rigorously adhered to Taylor and Betz’s (1983) classic five-dimensional theoretical framework. Researchers culturally adapted the original scale based on in-depth interview data from Chinese university students, ensuring the measurement tool possesses both an internationally recognized theoretical structure and alignment with career psychological characteristics within China’s collectivist culture. The scale comprises 39 items across five dimensions: self-evaluation, information gathering, goal selection, planning, and problem solving. Employing a five-point Likert scale, participants rate their confidence in completing stated tasks on a scale from “Not at all confident (1)” to “Very confident (5)”. Higher scores indicate stronger CDSE. Sample items include: “I can list several occupations or jobs that interest me” and “I can make career decisions without worrying whether they are right or wrong”. Its psychometric properties have been corroborated in multiple recent international studies using Chinese samples. Both A. Zhou et al. (2024) and Z. Liu and Liang (2025) explicitly employed Peng and Long’s (2001) scale to assess Chinese university students, reporting excellent psychometric indices. Recent evidence from culturally comparable samples further supports the scale’s structural stability and predictive validity within the Chinese context. In the present study, internal consistency was assessed using Cronbach’s α coefficient, yielding an α of 0.905. EFA revealed a KMO value of 0.906 and a Bartlett’s sphericity test *p*-value < 0.001, confirming the data’s suitability for factor analysis. Furthermore, the construct validity of the scale was demonstrated through CFA (χ^2^/df = 2.775, CFI = 0.949, TLI = 0.933, RMSEA = 0.066, SRMR = 0.034).

Parental Career Support: Parental career support was measured using the behavioral subscale (Support-B) of the Parental Career Support Scale refined by Leung and Zhou (2025). This 12-item scale comprises three dimensions: Action support; Autonomy affirming support; Emotional support. The scale employs a 5-point Likert format, requiring participants to rate statements on a scale from “Never (1)” to “Very often (5)” to indicate the frequency of parental career support behaviors. Higher scores indicate greater frequency of such behaviors. Example items include: “My parent showed me where I could find information about different universities and occupations,” “My parents encouraged me to choose any occupations that I am interested in,” and “My parents asked me about the occupations that I would consider in the future.” In this study, Cronbach’s α coefficient was used to assess the scale’s internal consistency, yielding a value of 0.932. EFA revealed a KMO value of 0.923 and a Bartlett’s sphericity test *p*-value < 0.001, confirming the data’s suitability for factor analysis. Furthermore, the scale underwent CFA, yielding high construct validity χ^2^/df = 2.833, CFI = 0.976, TLI = 0.967, RMSEA = 0.067, SRMR = 0.036.

The measurement limitations of this behavioral subscale—specifically its inability to distinguish support frequency from support quality—are discussed in the theoretical framework (see Section 2.4).

Career Decision-making Difficulties: CDD were measured using the Chinese version of the CDDQ, revised by Wu et al. (2016). This scale was developed based on the taxonomy theory of CDD proposed by Gati et al. (1996), which originally comprised 44 specific difficulty types. Wu et al. (2016) conducted cross-cultural adaptation and revision of the original version to align with the Chinese cultural context, ultimately producing a 36-item Chinese version. This scale has been extensively applied and validated among Chinese undergraduate and postgraduate students, demonstrating sound psychometric properties. The cross-cultural structure of the CDDQ has been validated across 13 countries using nine language versions (Levin et al., 2023). Bi et al. (2023) confirmed the scale exhibits exceptionally high internal consistency (Cronbach’s α = 0.933) among Chinese postgraduate students. The scale comprises three core dimensions: Lack of Readiness, Lack of Information, and Inconsistent Information. Participants responded using a 5-point Likert scale (1 = Strongly disagree, 5 = Strongly agree), with higher total scores indicating greater perceived CDD. In this study, Cronbach’s α was 0.812, demonstrating good internal consistency. EFA revealed a KMO value of 0.78 and a Bartlett’s sphericity test *p*-value < 0.001, confirming data suitability for factor analysis. Furthermore, CFA results were satisfactory χ^2^/df = 2.72, CFI = 0.97, TLI = 0.953, RMSEA = 0.065, SRMR = 0.038.

Validity Summary: In addition to the fit indices reported above, confirmatory factor analyses supported the convergent and discriminant validity of all multi-item constructs. Detailed indices, including Composite Reliability (CR), Average Variance Extracted (AVE), and Heterotrait–Monotrait ratios (HTMT), are systematically reported in the Results section.

##### Common Method Bias Mitigation

Given that all data were self-reported, procedural and statistical remedies were implemented to mitigate potential common method bias (CMB). Procedurally, two design-level strategies were adopted. First, participant anonymity was guaranteed throughout data collection to minimize social desirability responding. Second, scale format differentiation was employed: the Career Anxiety scale used a 4-point Likert format whereas all remaining scales employed a 5-point format. This deliberate variation in response format was intended to reduce consistency motif bias and response set tendencies by discouraging participants from applying a uniform response pattern across instruments (Podsakoff et al., 2003).

Statistically, considering the limitations of traditional diagnostic tools, we employed a dual-testing strategy. First, as an initial screening rather than a definitive test, Harman’s single-factor test was conducted. Results showed that the first principal component accounted for only 23.26% of the total variance, falling significantly below the critical 40% threshold. However, acknowledging that this technique has been criticized for its insensitivity (Podsakoff et al., 2003, 2012), we did not rely solely on this result.

Consequently, we prioritized the Unmeasured Latent Method Construct (ULMC) approach to conduct rigorous verification. As is common in self-reported datasets, the inclusion of a latent method factor yielded a noticeable improvement in global fit indices (ΔCFI = 0.056, ΔRMSEA = 0.025). While this suggests the presence of some shared method variance, the decisive criterion for CMB interference is parameter bias. 

To accurately quantify this impact, we compared the standardized factor loadings of all measurement items on their respective trait factors between the baseline model and the method-factor model. The results revealed that the trait factor loadings remained exceptionally robust, with an average absolute change (|Δλ|) of only 0.003, and no single item’s loading changing by more than 0.005. Importantly, the vast majority of loadings exceeded the recommended threshold of 0.50 (Hair et al., 2019), with the lowest loading being 0.49, which remains well within the acceptable bounds for construct retention and validity.

Furthermore, a comparative analysis demonstrated that the significance and direction of all core structural path coefficients remained invariant across both the trait-only and method-factor models. This parameter stability confirms that although method variance exists, it does not substantively distort the theoretical relationships or the validity of the study’s conclusions (Richardson et al., 2009).

#### 3.1.3. Statistical Analysis

Following contemporary best practices (Kline, 2023; Brown, 2015), this study adopted a hybrid analytical strategy that integrates the strengths of latent variable modeling for measurement validation and robust resampling-based regression for hypothesis testing. 

Measurement Model Evaluation. First, CFA was conducted using AMOS 26.0 to verify the convergent validity and composite reliability of the latent variables. This approach was supported by the established factor structures of the instruments used and allowed a balance between theoretical fidelity and analytical parsimony. Upon confirming the satisfactory factor structure (see Section 3.1.2), composite mean scores were computed for subsequent analyses. While we acknowledge that converting latent constructs to observable composite scores simplifies measurement error structures—which, as Cole and Preacher (2014) and Ledgerwood and Shrout (2011) caution, may introduce some degree of parameter estimation bias—this approach was adopted to leverage the robust bootstrapping capabilities of the PROCESS macro for testing complex moderated mediation. To mitigate potential bias, we ensured high measurement reliability (see Section 3.1.2) and verified the measurement model through CFA prior to path analysis, a strategy that has been applied in prior career development research.

Hypothesis Testing and Model Specification Strategy. To examine the proposed structural relationships, we employed PROCESS macro v4.1 for SPSS Model 5, which estimates an unconditional indirect association through a mediator and a moderated direct association between the predictor and outcome. This approach balances methodological rigor, parsimony, and interpretability. Although latent variable approaches, such as latent moderated structural equations (LMS) in Mplus, model measurement error explicitly, latent interaction estimation generally requires larger samples and may involve convergence difficulties and reduced power, particularly when interaction effects are modest (Hayes, 2018). Because this study focuses on theoretically specified conditional associations rather than complex latent interactions, PROCESS was considered appropriate and transparent. This approach also facilitates interpretation of conditional associations among established psychological constructs within the explanatory mixed-methods framework.

In addition, because the product of path coefficients (a × b) typically follows a non-normal and often skewed sampling distribution, traditional parametric tests of indirect effects, such as the Sobel test, may have limited statistical power and produce less accurate inferences (Shrout & Bolger, 2002). Therefore, regardless of the distributional characteristics of the raw data, we used a bootstrapping procedure with 5000 resamples to generate 95% bias-corrected confidence intervals (CIs). Effects were considered statistically significant when the corresponding confidence interval did not include zero.

To further enhance analytical rigor and avoid the inflation of familywise error rates that may arise from fragmented or stepwise testing, we did not conduct separate piecemeal analyses. Instead, we directly estimated the full conditional process model using Hayes’ PROCESS Model 5. The specification of this model was theoretically grounded in the Social Cognitive Model of Career Self-Management (Lent & Brown, 2013). Accordingly, the present analysis was conceptualized as a conditional process model rather than a fully moderated mediation model. Specifically, CDSE was specified as a statistical mediator representing the indirect association between career anxiety and CDD, while parental career support was specified as a moderator of the direct association between career anxiety and CDD. Thus, the indirect association through CDSE was estimated as an unmoderated mediation pathway, whereas the direct association between career anxiety and CDD was tested conditionally across different levels of parental career support.

### 3.2. Qualitative Phase

#### 3.2.1. Participants

The qualitative phase was designed to provide contextual interpretations of the moderation pattern identified in the quantitative phase. Specifically, the quantitative results showed that the anxiety–CDD association was stronger among students reporting higher parental career support frequency. Because H3 did not specify the direction of moderation, the interviews were used to explore participants’ subjective accounts that might help contextualize this empirically observed pattern. Following the explanatory sequential logic of mixed-methods research (Creswell & Plano Clark, 2018), we employed a theoretically informed purposeful sampling matrix (Palinkas et al., 2015; see Table 2) organized around four combinations of career anxiety and parental career support frequency: high anxiety/high support frequency, high anxiety/low support frequency, low anxiety/high support frequency, and low anxiety/low support frequency. This design was intended to help interpret the statistical interaction while allowing participants’ own accounts and emergent themes to inform the qualitative analysis.

The sampling logic was aligned with the simple-slope framework used in the quantitative analysis. Using mean ± 1 SD as the boundary criterion (Aiken & West, 1991), participants were classified into high and low groups in both career anxiety and parental career support frequency, yielding a 2 × 2 theoretical sampling matrix. Sample selection followed the combined principles of prioritizing theoretically relevant cases and seeking within-group thematic sufficiency. Because the moderation pattern was most directly relevant to participants with elevated career anxiety, Groups I and II were prioritized in initial recruitment and analytical attention. Groups III and IV each included three participants to provide systematic comparative material. In total, twelve participants were interviewed.

Given the small number of participants within each quadrant, saturation was not treated as a definitive endpoint, nor do we claim full thematic saturation within each subgroup. Instead, iterative coding was used to assess whether later interviews generated substantively new insights or mainly elaborated previously identified interpretive patterns. Across the twelve interviews, later interviews largely confirmed and refined earlier patterns, suggesting preliminary thematic stability across the full qualitative sample rather than complete saturation within each quadrant. This approach is consistent with methodological recommendations emphasizing iterative assessment of new information emergence during qualitative analysis (Francis et al., 2010) and prior evidence that focused, relatively homogeneous samples may achieve thematic sufficiency with relatively small numbers of interviews (Guest et al., 2006). Accordingly, the qualitative findings are interpreted as contextual and subjective accounts of participants’ experiences, not as evidence of mediating mechanisms or population-level explanations of the quantitative interaction. 

#### 3.2.2. Data Collection Procedure and Interview Protocol

Data were collected via semi-structured, in-depth interviews conducted between November and December 2025. Each interview lasted between 45 and 60 min and was audio-recorded with participants’ consent. To ensure the qualitative inquiry directly addressed the quantitative findings, an interview guide was developed centering on three core thematic blocks:

First, participants were asked about their lived experiences of career anxiety. Questions invited them to describe specific situations in which they felt anxious about future employment, postgraduate entrance examinations, civil-service examinations, job applications, or other career-related choices. Example questions included: “Can you describe a recent moment when you felt anxious about your future career?” and “What did you do after experiencing this anxiety?”

Second, participants were asked about their perceptions of parental career involvement. Questions focused on concrete forms of parental involvement, such as providing information, recommending jobs or examinations, discussing location choices, expressing expectations about stable employment, offering financial support, or commenting on career plans. Example questions included: “What specific things have your parents done or said regarding your career decisions?” and “How did you feel when they became involved in this way?” These questions were designed to distinguish the frequency of parental involvement from its perceived quality, fit, emotional tone, and autonomy support.

Third, participants were asked about their cognitive and emotional interpretations of parental involvement during career decision-making. This block explored how students made sense of parental involvement under conditions of anxiety, without assuming that parental support was necessarily helpful or harmful. Example questions included: “When you are anxious about career decisions, how do you usually experience your parents’ involvement?” and “Can you describe what goes through your mind when your parents offer advice or support?” Follow-up probes allowed participants to discuss issues such as filial responsibility, economic reliance, expectations for stable employment, geographic mobility, guilt, autonomy, and perceived mismatch between parental advice and personal goals.

This semi-structured format allowed the researchers to maintain focus on the study’s core constructs while remaining open to emergent themes, including participants’ descriptions of guilt, obligation, resource mismatch, autonomy support, and relational expectations.

#### 3.2.3. Data Analysis and Coding Procedure

Qualitative data analysis followed Braun and Clarke’s (2006) six-stage thematic analysis framework and incorporated a hybrid coding strategy combining deductive and inductive approaches. The analysis aimed to explore participants’ subjective interpretations of the patterns identified in the quantitative model. Deductive attention was given to the study’s core constructs, including career anxiety, CDD, CDSE, and parental career support frequency. At the same time, inductive coding was used to identify themes emerging from participants’ own accounts, such as perceived resource mismatch, guilt associated with parental expectations, autonomy constraints, and perceived differences between support frequency and support quality.

The coding process comprised three steps.

Phase One: Independent Coding and Codebook Development. To ensure coding reliability, a coding team comprising two researchers trained in qualitative research was established.

Initially, both coders randomly selected 20% of interview transcripts for pre-coding, identifying recurrent semantic units. Following team discussion, an initial “coding dictionary” was established, defining each node’s scope and boundaries.

Subsequently, the two coders conducted independent line-by-line coding of the remaining material in parallel, guided by this dictionary. Throughout this process, coders maintained theoretical sensitivity to quantitative outcomes (e.g., “anxiety,” “parental career support”) while remaining receptive to novel concepts emerging from the data.

Phase Two: Consistency Testing and Consensus Building. Upon completion of coding, node comparisons were conducted using NVivo 12.0 software. Calculations revealed a Cohen’s Kappa inter-rater reliability coefficient of 0.81 for core nodes, indicating high coding reliability. For coding discrepancies (e.g., classifying a parental behavior as “autonomy-supportive” versus “controlling”), a third senior researcher was introduced to conduct “consensus meetings” until 100% consensus was achieved.

Phase Three: Thematic Hierarchy Extraction. Analysis followed a three-tiered extraction logic: “Open Coding (Tier 1) → Descriptive Sub-themes (Tier 2) → Analytical Core Themes (Tier 3)” (see Table 3):

## 4. Results

### 4.1. Quantitative Findings

#### 4.1.1. Measurement Model Evaluation and Descriptive Statistics

1. Descriptive Statistics and Correlation Matrix

Before testing the structural relationships, preliminary checks confirmed that the data quality met the requirements for subsequent analyses. As shown in Table 4, descriptive statistics indicated acceptable univariate normality (skewness < |3|, kurtosis < |10|; Kline, 2023). Table 4 also presents the means, standard deviations, and bivariate correlations among the core variables, providing preliminary evidence consistent with the hypothesized relationships.

2. Measurement Model Fit and Reliability/Validity

To rigorously evaluate the measurement model, this study conducted CFA and reliability testing for the four latent variables: career anxiety, CDSE, parental career support, and CDD. Cronbach’s alpha coefficients for all latent variables ranged from 0.792 to 0.932, while CR values ranged from 0.901 to 0.957, exceeding the recommended threshold of 0.70. AVE values were consistently above 0.50, indicating sound reliability and convergent validity across scales.

3. Discriminant Validity and Common Method Bias

Discriminant validity tests (see Table 5 diagonal) revealed that the square root of each variable’s AVE exceeded its correlation coefficients with other variables. Furthermore, all HTMT (Heterotrait–Monotrait ratio) indicators were below 0.85, robustly confirming the statistical and conceptual independence and discriminant validity of each construct. Alongside these validity checks, rigorous diagnostic procedures confirmed that CMB was effectively controlled and did not systematically distort the data structure (see Methodology (Section Common Method Bias Mitigation) for detailed CMB mitigation strategies).

#### 4.1.2. Correlation Analysis

Table 4 reveals that career anxiety exhibits a significant positive correlation with CDD (*r* = 0.460, *p* < 0.001) and a significant negative correlation with CDSE (*r* = −0.275, *p* < 0.001). CDSE shows a significant negative correlation with CDD (*r* = −0.286, *p* < 0.001). Parental career support exhibited a significant negative correlation with career anxiety (*r* = −0.281, *p* < 0.001), a significant positive correlation with CDSE (*r* = 0.429, *p* < 0.001), and a significant negative correlation with CDD (*r* = −0.268, *p* < 0.001). While these zero-order correlations suggest a generally beneficial role of support, it is critical to note that such bivariate associations may mask the conditional nature of support effects under specific contexts (e.g., high anxiety arousal), which will be further disentangled in the subsequent moderation analysis.

**Table 4:** Descriptive Statistics and Correlation Matrix.

Variables	*M*	*SD*	Skewness	Kurtosis	1	2	3	4
CA	2.703	0.45	−0.136	0.651	—	—	—	—
2. CDSE	3.636	0.56	−0.235	2.083	−0.275 ***	—	—	—
3. PCS	3.517	0.75	−0.508	0.630	−0.281 ***	0.429 ***	—	—
4. CDD	3.035	0.60	−0.418	0.632	0.460 ***	−0.286 ***	−0.268 ***	—

Note. *N* = 407. CA = Career Anxiety; PCS = Parental Career Support. *** *p* < 0.001.

**Table 5:** Convergent and Discriminant Validity (Fornell–Larcker Criterion and HTMT Ratios).

Variables	CR	AVE	1	2	3	4
CA	0.947	0.599	**(0.774)**	0.325	0.327	0.574
2. CDSE	0.949	0.557	—	**(0.746)**	0.467	0.334
3. PCS	0.957	0.655	—	—	**(0.809)**	0.308
4. CDD	0.901	0.518	—	—	—	**(0.720)**

Note. *N* = 407. Diagonal values in bold parentheses are the square root of the AVE for each construct. Values above the diagonal are Heterotrait–Monotrait (HTMT) ratios.

#### 4.1.3. Mediation Analysis

The mediating role of CDSE between career anxiety and CDD was examined using Model 4 of the PROCESS macro. To facilitate effect size comparison, all regression coefficients are reported as both unstandardized (*B*) and standardized (*β*) estimates. Detailed results are presented in Table 6.

**Figure 2:**
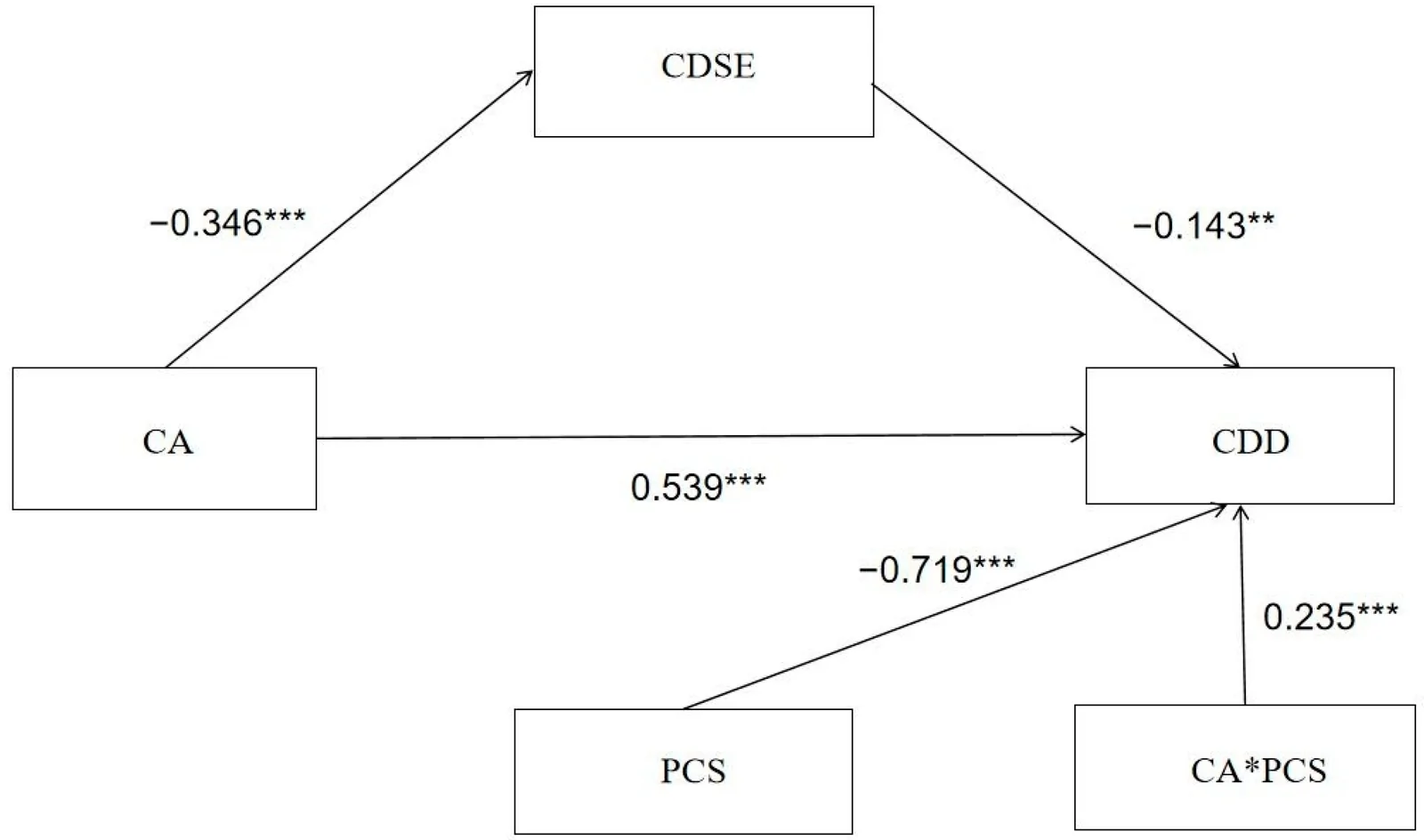
Statistical Diagram of the Conditional Process Model. Note. Values represent unstandardized coefficients (*B*). Control variables (grade level, discipline, and only-child status) are included in all regression models but omitted from the figure for clarity. ** *p* < 0.01, *** *p* < 0.001.

The regression results revealed a significant negative association in the first stage of the mediation path (Path a): career anxiety was significantly negatively associated with CDSE (*B* = −0.346, *β* = −0.275, *SE* = 0.060, *p* < 0.001). In the second stage (Path b), CDSE was significantly negatively associated with CDD (*B* = −0.183, *β* = −0.173, *SE* = 0.048, *p* < 0.001). After controlling for the mediating mechanism of CDSE, the direct effect of career anxiety on CDD (Path c’) remained robust and positive (*B* = 0.549, *β* = 0.412, *SE* = 0.060, *p* < 0.001). A statistical diagram of this mediation relationship is presented in Figure 2.

Regarding the effect size, the bootstrap analysis confirmed a significant partial mediation effect, with an unstandardized indirect effect of 0.063 (95% CI [0.016, 0.087]) and a completely standardized indirect effect of 0.048. To rigorously quantify the effect magnitude, we calculated the proportion of the mediated effect (P_M_ = ab/c). The indirect effect via CDSE accounted for 10.3% of the total effect (P_M_ = 0.103), with a 95% bootstrap CI [0.026, 0.142] excluding zero, confirming significant but partial mediation. The dominance of the direct effect (accounting for 89.7% of the total effect) suggests that under heightened contextual pressure, anxiety may operate through pathways not fully captured by CDSE alone, a pattern further illuminated in the qualitative phase (see Section 4.2). These results indicate that while the efficacy-based pathway is significant, a substantial proportion of the association between career anxiety and CDD remains direct. Thus, Hypothesis 2 was supported. 

#### 4.1.4. Testing the Conditional Process Model

This study employed Model 5 within the IBM SPSS PROCESS macro v4.1 to examine the conditional process model. The interaction term between career anxiety and parental career support frequency yielded a significant positive coefficient (*B* = 0.235, *SE* = 0.062, *t* = 3.79, *p* < 0.001) (see Table 7). This product term indicates that the positive association between career anxiety and CDD varied significantly across levels of parental career support frequency. Its substantive pattern is best interpreted through conditional associations, namely simple slope analysis. Notably, the addition of the interaction term explained an additional 2.6% of the variance in decision difficulty (Δ*R*^2^ = 0.026, F_change_(1, 402) = 14.36, *p* < 0.001). It is important to acknowledge that this increment in explained variance is modest in absolute terms. Nevertheless, this magnitude is consistent with the typical size of interaction terms detected in observational field studies, where product terms are routinely attenuated by measurement error, range restriction, and multicollinearity (Aguinis et al., 2005). Therefore, the moderation pattern is interpreted cautiously and in conjunction with the simple slope results. 

The positive interaction coefficient indicates that parental career support strengthens the positive association between career anxiety and CDD. This pattern suggests that under conditions of elevated anxiety, additional parental involvement may not serve a buffering role and may be associated with greater perceived decision-related burden. 

**Figure 3:**
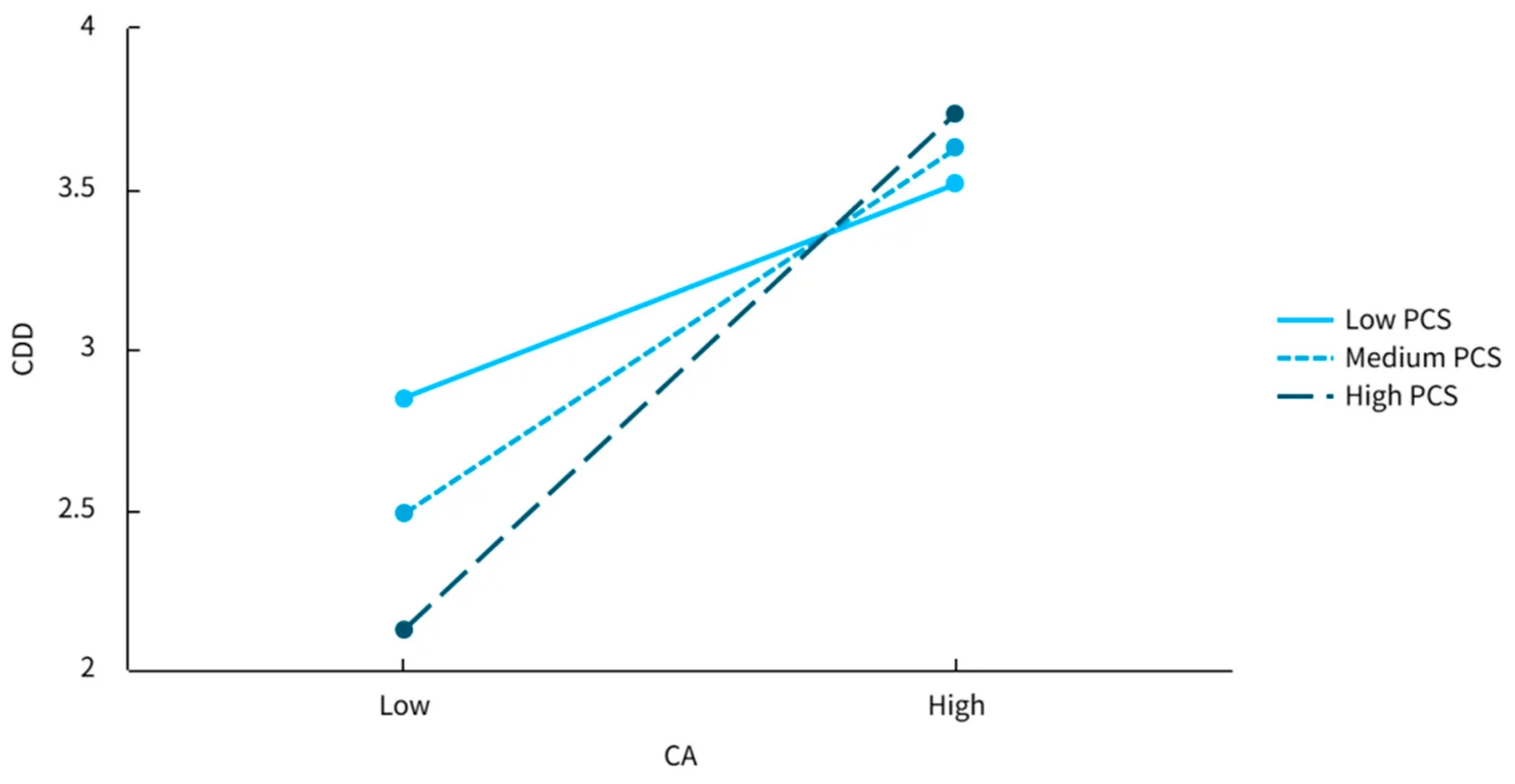
Simple slope plot illustrating the moderating role of parental career support in the association between career anxiety and CDD.

Simple slope analyses further revealed the pattern of moderation (see Table 8 and Figure 3). At low levels of parental career support frequency (−1 *SD*), career anxiety was positively associated with CDD, but the association was relatively weaker (simple slope = 0.363, *t* = 4.93, *p* < 0.001). In contrast, at high levels of parental career support frequency (+1 *SD*), this association was stronger (simple slope = 0.715, *t* = 9.16, *p* < 0.001). Thus, higher parental career support frequency was associated with a steeper positive anxiety–CDD association. The significant interaction therefore supported Hypothesis 3. The positive direction of the interaction indicated that the association between career anxiety and CDD was stronger at higher levels of parental career support frequency. Because H3 was formulated as a non-directional moderation hypothesis, this strengthening pattern was interpreted as an empirically observed result rather than an a priori directional prediction.

### 4.2. Qualitative Contextual Interpretations of the Quantitative Findings

Consistent with the explanatory sequential design, the qualitative phase was used to provide contextual interpretations of the statistical relationships observed in the quantitative model. Thematic analysis was conducted through dual-coder independent coding (Cohen’s Kappa = 0.81), iterative refinement of the codebook, and ongoing monitoring of thematic sufficiency. The three themes reported below were derived from recurrent cross-case patterns across multiple participants and theoretical subgroups, rather than from isolated illustrative quotations alone.

Taken together, the qualitative findings provide situational, cognitive, and relational-contextual interpretations of the associations estimated in the conditional process model. These themes should be read as participants’ subjective accounts of how they experienced career anxiety, decision difficulty, and parental involvement, rather than as evidence of causal mechanisms, mediating processes, or population-level explanations of the quantitative interaction.

Theorem 1: Structural Constraint as Lived Experience: When Opportunity Scarcity Becomes Decision Paralysis.

Theme One provides contextual interpretation of the strong positive association between career anxiety and career decision-making difficulties identified in the quantitative model (H1/direct pathway). Across multiple cases, anxiety was described not as a diffuse or irrational emotional reaction, but as a response to a career environment perceived as increasingly narrow, competitive, and structurally constrained.

A recurring pattern was that decision difficulty arose not from an overabundance of options, but from the perceived contraction—and in some cases near disappearance—of viable opportunities. Participant 5, for example, noted that “fewer and fewer primary school teaching positions are being recruited now,” while Participant 3 described teacher establishment positions as “very few,” leaving graduates with “very limited opportunities even to apply.” Similarly, Participant 2 reported that in her home province, positions aligned with her major were “almost nonexistent,” reducing her choices to a very narrow range of less specialized alternatives.

Across these accounts, anxiety was closely tied to the experience of constrained opportunity structures. Career decision difficulty appeared to emerge less from generalized indecisiveness than from trying to make choices within a field experienced as already restricted. Participants described delaying applications, narrowing their search prematurely, and struggling to evaluate alternatives meaningfully because the perceived option set was too limited to sustain confident exploration. This pattern helps contextualize the quantitative finding that higher career anxiety was associated with greater career decision-making difficulties: for many participants, anxiety and difficulty were embedded in the lived experience of structural blockage rather than in individual decisional weakness alone.

Theorem 2: The Psychology of “Zero Tolerance for Error”: Anticipated Risk and Fragile Self-Efficacy.

Theme Two provides contextual interpretation of the indirect association involving CDSE in the relationship between career anxiety and decision difficulty (H2). Across several cases, anxiety did not appear to diminish participants’ sense of underlying ability per se; rather, it altered how they evaluated action, uncertainty, and the possible consequences of making mistakes.

Participants described a cognitive orientation that may be characterized as a “zero tolerance for error” frame. Within this frame, ordinary exploratory behaviors—such as applying for a position, testing an option, or revising a plan—were no longer experienced as normal steps in career development, but as high-stakes actions carrying potentially irreversible consequences. Participant 4 stated that “the tolerance for error… is far too low,” suggesting that even minor missteps could mean losing “a very important opportunity.” Participant 5 similarly reported that anxiety made her “not even dare to apply” for certain positions, while Participant 1 described being “very afraid of making the wrong decision” despite not viewing herself as incapable.

Although relational expectations sometimes intensified this pattern, participants’ accounts primarily reflected internal cognitive and emotional processes associated with anxiety and decision-making. Several participants described heightened sensitivity to the possible consequences of failure, such that the perceived cost of error became so great that confidence in taking exploratory action was weakened. Under these conditions, participants became more likely to second-guess themselves, delay commitment, or avoid potentially informative action altogether.

In sum, these accounts suggest that anxiety was associated with reduced CDSE not simply because participants perceived themselves as lacking competence, but because they became less willing to trust their own judgment within a career context experienced as unforgiving. This provides a qualitative interpretation of the quantitative association between higher career anxiety and lower CDSE: participants described self-efficacy as becoming more fragile when career-related actions were perceived as risky.

This pattern was observed in a majority of participants (e.g., 8 out of 12), particularly among those in the high-anxiety subgroup, with illustrative examples drawn from Participants 1, 4, and 5. 

Theorem 3: Conditionality of Parental Career Support: Resource Mismatch, Autonomy Constraint, and Contrasting Patterns of Involvement.

Theme Three provides contextual interpretations of the moderation pattern observed in the quantitative model (H3), whereby parental career support strengthened rather than buffered the positive association between career anxiety and decision difficulty. Across multiple cases, participants’ accounts suggested that the function of parental involvement depended less on how frequently parents were involved than on whether that involvement was experienced as relevant, autonomy-supportive, and responsive to students’ needs under anxiety.

1. Resource mismatch and cognitive overload

One recurrent pattern involved a mismatch between parental input and students’ decision contexts. Several participants described receiving large amounts of job-related information from parents, but much of it was experienced as unscreened, poorly targeted, or unrelated to the student’s qualifications and career direction. Participant 5 referred to receiving “a lot of messy recruitment information,” while Participant 2 noted that many of the positions her parents forwarded were ones she “could not even apply for.”

Rather than reducing uncertainty, this kind of involvement often increased cognitive burden. Participants described having to sort through irrelevant information while already feeling overwhelmed, which in turn intensified anxiety and made decision-making feel more confusing rather than more manageable. In this sense, parental involvement that was high in volume but low in contextual fit was often experienced less as a resource than as an additional demand on already strained cognitive capacities.

2. Autonomy constraint within emotionally supportive involvement

A second recurring pattern concerned the blurring of support and constraint within emotionally warm parent–child relationships. Some participants described parental expectations—such as remaining geographically close to home, pursuing stable occupations, or prioritizing examination-based pathways—as being communicated through care and concern rather than overt pressure. Yet these expectations nonetheless shaped the boundaries of what participants felt able to choose.

For Participants 1 and 9, for example, parental involvement was not described as explicitly coercive; rather, it operated through subtle relational pressure. Because these expectations were framed as protective or well-intentioned, they were difficult to reject without experiencing guilt or a sense of disobedience. Under these conditions, support became psychologically ambiguous: it was received as care, but also experienced as narrowing the range of acceptable options. This ambiguity appeared particularly consequential when participants were already highly anxious, because the added relational weight made autonomous decision-making more difficult.

3. Autonomy-supportive involvement as a contrasting pattern

At the same time, the qualitative data did not support a blanket interpretation that parental career support is inherently problematic. Contrasting cases showed that parental involvement could remain beneficial when it preserved students’ decisional autonomy and responded selectively to their needs.

For example, Participant 8 described a pattern of autonomy-supportive involvement, explaining that although her parents offered suggestions, “the final decision was still mine,” and that “whatever I decide, they support me.” Similarly, Participants 4 and 10 reported that parental support reduced rather than intensified anxiety when it took the form of emotional reassurance without imposing a preferred direction. In these cases, parental involvement appeared to function in a manner more consistent with the conventional stress-buffering interpretation.

A further contrasting pattern emerged in the high-anxiety–low-support group. Participant 7 described limited parental involvement as creating space for independent reflection, allowing her to consider options without external interference. This case suggests that lower parental involvement does not necessarily intensify difficulty when reduced involvement preserves psychological room for autonomous exploration.

Taken together, these contrasting accounts indicate that the moderation effect observed in the quantitative model should not be interpreted as evidence that parental support is detrimental per se. Rather, the qualitative findings suggest that the effects of parental involvement depend on its quality, selectivity, and autonomy-supportiveness. Under conditions of heightened anxiety, more intensive parental involvement appeared more likely to be experienced as interference-like when high involvement coincided with low autonomy support or poor contextual fit, whereas involvement experienced as autonomy-supportive retained a more clearly facilitative function.

4. Integrative Interpretation

Taken together, the qualitative findings add interpretive depth to the conditional process model by showing that career decision-making difficulties were shaped not only by individual anxiety, but also by the perceived structure of opportunity, the anticipated cost of error, and the relational quality of parental involvement. In this way, the qualitative phase clarifies how structural, cognitive, and relational processes jointly help contextualize the statistical associations observed in the quantitative analysis. 

## 5. Discussion

This study used an explanatory sequential mixed-methods design to examine cross-sectional associations among career anxiety, CDSE, parental career support frequency, and CDD among female undergraduates from four purposively selected non-elite universities in Jiangsu Province. Three interpretive threads structure the discussion: the concurrent association between career anxiety and CDD, the indirect association involving CDSE, and the conditional association between career anxiety and CDD across different levels of parental career support frequency.

### 5.1. The Contextualized Nature of Career Anxiety

The robust positive association between career anxiety and CDD extends beyond individual emotional vulnerability; it coincides with participants’ shared perceptions of constrained opportunities. While this pattern echoes prior correlational research documenting that anxiety co-occurs with impaired career decision-making experiences (Mata-Correas et al., 2025; Udayar et al., 2020), the present study places this association within the unique institutional and cultural landscape of Chinese higher education and family systems.

Our qualitative findings suggest that many participants viewed state-sector employment as the most viable pathway under conditions of uncertainty. This pattern is consistent with prior research showing that state-sector employment in China correlates with greater protection against economic shocks (Qian & Fan, 2020) and with earlier work showing the historically strong role of state-linked institutions in urban job allocation (cf. Bian, 1994). In this sense, career anxiety appears less as an individual deficit than as a subjective outlook that coexists with a constrained opportunity structure.

This state-sector orientation reflects a form of bounded rationality (Simon, 1955): faced with incomplete information and high uncertainty, these students adopt state-sector employment as a cognitive anchor—a satisficing heuristic that reduces decision complexity by narrowing the feasible choice set. In contemporary Chinese families, this preference is often reinforced by intergenerational expectations that prioritize employment stability and social security, particularly through state-sector positions. For female undergraduates navigating perceived constraints related to university tier stratification and gendered labor-market barriers, prioritizing state-sector employment correlates with perceived disadvantages in competitive private-sector recruitment. This interpretive framing aligns with prior work noting state-sector roles are broadly perceived as a buffer against career instability within China’s job market.

However, this rational orientation also co-occurs with elevated self-reported decision paralysis when participants perceive limited openings in these coveted fields. As the qualitative findings illustrate, when access to this highly competitive pathway appears limited, this occupational preference aligns with heightened self-reported decision paralysis. In this way, career anxiety emerges not as an individual deficit, but as a subjective experience that correlates with structurally restricted choice environments. 

### 5.2. Career Decision-Making Self-Efficacy as a Partial Correlational Pattern Under Heightened Pressure

Although CDSE exhibited a statistically significant partial indirect association (H2 supported), the relatively small share of the total cross-sectional association accounted for by this indirect pathway (10.3%) alongside the dominant direct pairwise correlation (*β* = 0.412 vs. *β* = 0.048) suggests that the association involving CDSE appears to vary depending on contextual conditions. While SCCT posits that affective states share correlational ties with self-efficacy perceptions and in turn align with career behavioral tendencies (Lent et al., 1994), the present cross-sectional patterns indicate that this cognitive linkage may weaken under conditions of elevated perceived environmental threat.

Rather than proposing a novel threshold construct, we interpret this pattern through established cognitive and motivational theories. First, the finding is broadly consistent with the Yerkes–Dodson framework, which suggests that excessive psychological arousal may interfere with effective cognitive performance, such that self-reported excessive anxiety coincides with less effective cognitive functioning. In this context, cumulative structural and career-related pressures may coincide with levels of anxiety that interfere with effective cognitive processing, which correlates with weaker self-efficacy-based career regulation.

Second, Attentional Control Theory (ACT; Eysenck et al., 2007) illuminates the proximal cognitive correlates behind this pattern. ACT proposes that anxiety is associated with a shift from goal-directed to stimulus-driven cognitive processing, which may correspond to reduced executive efficiency. In the present study, qualitative evidence indicates participants with high anxiety display persistent vigilance toward threat-related cues (e.g., shrinking employment opportunities, institutional barriers), which correlates with depleted cognitive bandwidth for planning, evaluation, and decision-making. As CDSE fundamentally relies on these executive functions, lower self-efficacy perceptions are associated with greater career decision-making difficulties under high anxiety within this cross-sectional sample.

Taken together, these concurrent associations demonstrate CDSE’s correlational linkage with career decision outcomes is not universally stable, but context-dependent. Under moderate conditions, self-efficacy perceptions align with adaptive career planning behaviors. However, under structurally constrained and high-anxiety environments, affective strain coexists with weaker reliance on self-efficacy-related cognitive processing, and anxiety remains strongly associated with decision difficulties in this sample. This interpretation remains consistent with SCCT, which does not assume that efficacy-related processes exert uniform influence across all contexts. The theory does not assert that the efficacy pathway will dominate under all conditions. Rather, the present findings enrich SCCT by specifying a boundary condition under which this intervening linkage becomes attenuated—namely, when perceived environmental threat is sufficiently severe to operate beyond the explanatory contribution of efficacy-related processes.

### 5.3. The Conditional Correlational Role of Parental Career Support

One of the most theoretically noteworthy cross-sectional patterns observed is that higher self-reported frequency of parental career support behaviors was associated with a stronger positive conditional association between career anxiety and CDD, rather than a weaker linkage. This pattern should be interpreted in light of the way parental support was measured. The quantitative measure captured the frequency of supportive behaviors rather than their perceived quality, autonomy-supportiveness, or contextual fit. Therefore, the finding reflects differences in the association between support frequency and decision difficulties rather than the effect of parental support itself.

Instead, the qualitative findings provided contextual interpretations of how frequent parental career involvement may be experienced alongside heightened decision strain under conditions of severe career anxiety. Specifically, participants described situations in which parental input was perceived as mismatched with their actual circumstances or created emotional difficulty in rejecting advice due to feelings of guilt or obligation. In such cases, frequent supportive behaviors appeared to coexist with subjective experiences resembling interference rather than being experienced exclusively as protective resources. This interpretation is consistent with Dietrich and Kracke’s (2009) distinction between parental support and parental interference as separate but potentially co-occurring dimensions, as well as Parola and Marcionetti’s (2022) argument that the developmental value of parental involvement depends on its qualitative characteristics rather than behavioral frequency alone.

This pattern differs from the predominantly buffering role of parental support documented in previous research and therefore warrants careful contextual interpretation. In contemporary Chinese families, parental career involvement and frequent supportive behaviors are culturally normative; however, such involvement may also coexist with implicit expectations regarding occupational stability, particularly preferences for state-sector employment. Within this cultural context, frequent parental career-related communication may sometimes reinforce pressure toward highly valued but competitive occupational pathways, such as civil service or teaching positions. This combination of high parental investment and strong expectations for stable employment may make frequent support more likely to be experienced as pressure rather than protection among students already facing substantial career anxiety and perceived employment constraints.

The qualitative data further highlighted two interrelated subjective experiences: resource mismatch and autonomy constraint. Participants described increased cognitive burden when parental information did not align with their qualifications, interests, or decision contexts. Some also experienced parental expectations as relational obligations that complicated independent decision-making and reduced perceived autonomy. These observations complement Guay et al. (2003), who demonstrated that parental behavioral styles are associated with career indecision through autonomy and self-efficacy-related processes, and X. Tian et al. (2021), who showed that parenting orientations are linked with career decision-making difficulties among Chinese college students through core self-evaluation and career calling.

A related subjective pattern identified in this study may be tentatively described as warmth-laden constraint: a form of involvement in which emotional care coexists with implicit boundaries surrounding acceptable career choices. This term is proposed as an inductive interpretive label rather than a validated construct. It differs from overt helicopter parenting, which previous research has associated with reduced autonomy, lower self-efficacy, and increased fear of failure (Park et al., 2025). Importantly, the qualitative findings do not suggest that parental career support is universally associated with decision strain. Previous research indicates that parental involvement can facilitate adaptive career development when embedded in supportive relational processes and accompanied by students’ psychological resources (Leung & Zhou, 2025). Taken together, the present cross-sectional findings support a support–context fit perspective: the implications of parental career involvement depend not simply on how frequently support behaviors occur, but on whether such involvement is experienced as relevant, autonomy-affirming, and compatible with students’ psychological needs under conditions of stress.

The interpretation of these findings should remain bounded by the characteristics of the sampled population. Specifically, the present study does not claim that the observed relationships represent all Chinese university students or all female undergraduates in non-elite institutions. Rather, the findings describe how career anxiety, parental career support, and decision-making difficulties are associated among female undergraduate students from four purposively selected non-elite universities in Jiangsu Province. Replication studies involving diverse institutional types, geographic regions, and student populations are required before broader generalization can be considered.

## 6. Limitations and Future Directions

Despite the contributions of this mixed-methods study, several limitations should be acknowledged.

First, an important limitation concerns the operationalization of parental career support, as this issue bears directly on the interpretation of the moderation finding (H3). The quantitative measure used in this study primarily captured the frequency of supportive parental behaviors rather than their qualitative character. As the qualitative findings subsequently revealed, however, support quality—and specifically whether parental involvement is experienced as autonomy-supportive, intrusive, controlling, or mismatched to students’ psychological needs—may be more consequential than behavioral frequency alone. This creates a conceptual gap between the quantitative operationalization and the theoretical interpretation advanced in the discussion. Accordingly, the observed moderation effect should be interpreted with caution: it may reflect not simply “more support,” but more frequent involvement that is experienced as psychologically burdensome under conditions of heightened anxiety. Future research should therefore adopt multidimensional measures that distinguish at least three components: support quantity, support quality, and perceived fit between parental involvement and the student’s autonomy needs.

Importantly, the qualitative themes should be interpreted as plausible subjective interpretations of how students experience parental involvement rather than as empirically established mediating mechanisms explaining the quantitative moderation effect. Because support quality, autonomy support, and perceived fit were not directly measured in the quantitative phase, these qualitative insights cannot determine whether such factors statistically account for the observed interaction or whether they generalize beyond the present sample.

Second, the cross-sectional nature of the quantitative design limits causal inference. Although the proposed pathways are theoretically grounded in Social Cognitive Career Theory and broadly consistent with the data, the present analyses capture associations at a single time point rather than developmental or temporal processes. Career anxiety, self-efficacy, and parental involvement are all likely to fluctuate across different phases of the job-search process. Future research should move beyond conventional longitudinal designs by employing ecological momentary assessment (EMA) or short-term intensive longitudinal designs to track how anxiety and support interactions evolve in real time during key career events—such as exam registration, application deadlines, interview periods, and family discussions about employment choices. Such designs would make it possible to identify more precisely when parental involvement functions as a buffer and when it begins to operate as pressure.

Third, the conditional process model was theoretically focused rather than exhaustive. Parental career support was modeled as moderating only the direct path from career anxiety to career decision-making difficulties—the path most central to the study’s core inquiry. This left two plausible conditional indirect processes untested: parental support may also moderate the anxiety → self-efficacy path, or the self-efficacy → decision-making difficulties path. The present model should therefore be understood as one theoretically motivated representation rather than a comprehensive account. Future studies should explicitly compare competing moderated mediation structures and use model-comparison indices to identify where in the career decision-making process parental involvement exerts its strongest conditional influence.

Fourth, all focal variables were measured through student self-report alone, with no parent-report data collected. Without dyadic data, it remains impossible to determine whether the observed interference effect reflects actual parental overinvolvement, perceptual mismatch between parent intentions and child experience, or both. Future research should employ parent–child dyadic designs in which both parties independently report on supportive behaviors, autonomy support, and decision-related pressure—allowing direct modelling of perceptual gaps and more precise attribution of the “warmth-laden constraint” dynamic identified here.

Fifth, the sample was intentionally context-specific. By focusing on female undergraduates in non-elite local universities, the study prioritized contextual depth over broad statistical generalizability. The findings should therefore not be generalized to all Chinese university students without caution. At the same time, this bounded focus may offer useful theoretical purchase for other settings characterized by strong intergenerational interdependence, gendered labor-market expectations, and high value placed on state-sector security. Future research could strengthen external validity through cross-institutional and cross-regional comparative sampling—for example, by comparing students from elite and non-elite universities, urban and non-urban regions, or disciplines with markedly different employment structures. Such designs would help clarify whether the interpretive patterns identified here—establishment-oriented logic, guilt-laden support, and warmth-laden constraint—are context-specific or more broadly transferable across institutional and cultural configurations.

Overall, these limitations point toward a more precise agenda for future research. Subsequent studies should move beyond single-wave and single-informant designs by combining multidimensional measurement of parental career support, parent–child dyadic data collection, and real-time or intensive longitudinal methods. Equally important, future work should systematically test competing moderated mediation structures—examining whether parental involvement conditions the anxiety–efficacy link, the efficacy–decision difficulty link, or both—across varied institutional and cultural contexts. Such an agenda would substantially clarify the conditions under which parental involvement operates as a buffer, a burden, or a context-contingent combination of both. 

## 7. Conclusions

Within the context of female undergraduates from four purposively selected non-elite universities in Jiangsu Province, this study shows that career decision-making difficulties are associated with both individual psychological processes and relational contexts. Career anxiety was positively associated with CDD, partly through reduced CDSE, while parental career support frequency altered the strength of this association. Importantly, the findings do not suggest that parental involvement is inherently harmful; rather, they indicate that the implications of support depend on how it is experienced, particularly regarding autonomy and fit with students’ career needs. Future research using longitudinal, dyadic, and cross-context designs is needed to clarify how parental involvement functions across different career development conditions.

## Figures and Tables

**Table 1:** Characteristics of the Participating Institutions and Sampling Rationale.

Code	Geographic Region	Institutional Type	Discipline	Sampling Rationale
University A	Southern Jiangsu (Developed Regional Context)	Regional University (Non-“Double First-Class”)	Polytechnic	Located in a developed economic region characterized by strong labor-market competition and higher living costs.
University B	Central Jiangsu (Intermediate Regional Context)	Regional University (Non-“Double First-Class”)	Comprehensive	Represents a transitional regional context with diverse disciplinary offerings, providing a reference point between more developed and less-developed settings.
University C	Northern Jiangsu (Less-developed Regional Context)	Regional University (Non-“Double First-Class”)	Normal/Teacher Education	Reflects a teacher-education-oriented context where public-sector employment pathways are particularly salient.
University D	Northern Jiangsu (Less-developed Regional Context)	Regional University (Non-“Double First-Class”)	Polytechnic	Provides a basis for comparison with University A by examining a similar disciplinary orientation within a different regional context.

Note. The geographic stratification (Southern, Central, and Northern Jiangsu) is operationalized based on regional economic aggregates and resource endowment levels. Specifically, Southern Jiangsu (Sunan) is anchored as the high-resource baseline, with a Regional GDP of 7.78 trillion RMB in 2024, according to the Jiangsu Statistical Yearbook 2025 (Jiangsu Provincial Bureau of Statistics, 2025). Quantitative analyses based on this purposive sample are context-specific and explanatory, examining whether the observed associations are robust across these environments rather than inferring national population parameters.

**Table 2:** Sampling Matrix and Characteristics of Interview Participants (*N* = 12).

Quadrant Grouping	Sampling Combination	Sample Size (*N*)	Filtering Criteria	Qualitative Inquiry Purpose
Group I	High Anxiety + High Support Frequency	3	Anxiety > *M* + 1*SD* AND Support Frequency > *M* + 1*SD*	Core Interpretive Focus: To explore how high-frequency parental career support behaviors may be experienced as burdensome under conditions of elevated anxiety, and to identify the psychological processes through which students interpret “well-intentioned actions” as mismatched, pressuring, or difficult to navigate, regardless of parental intent.
Group II	High Anxiety + Low Support Frequency	3	Anxiety > *M* + 1*SD* AND Support Frequency < *M* − 1*SD*	Comparative context: To examine how students with elevated anxiety described career decision-making experiences when parental career involvement was reported as infrequent, thereby providing contrastive material for interpreting the role of support frequency.
Group III	Low Anxiety + High Support Frequency	3	Anxiety < *M* − 1*SD* AND Support Frequency > *M* + 1*SD*	Comparative context: To examine how students with lower anxiety described frequent parental career involvement, including whether similar parental behaviors were interpreted differently under lower-anxiety conditions.
Group IV	Low Anxiety + Low Support Frequency	3	Anxiety < *M* − 1*SD* AND Support Frequency < *M* − 1*SD*	Background comparison: To describe career decision-making experiences among students reporting both lower anxiety and lower parental career support frequency, providing additional contextual material for interpreting cross-quadrant differences.

Note. *M* = Mean; *SD* = Standard Deviation. Criteria were based on the statistical distribution of the full quantitative sample. Parental career support in the sampling matrix refers to reported support frequency rather than support quality, autonomy support, or subjective satisfaction with support.

**Table 3:** Thematic Analysis Coding Framework: Examples of Codes, Sub-themes, and Core Themes.

Raw Data Excerpt (Representative Quotes)	Level 1 Coding: Open Coding	Level 2 Coding: Descriptive Sub-Themes	Level 3 Coding: Core Themes
[Participant 2]: “It’s not that I’m indecisive… but that primary education positions simply aren’t available… This reality of having ‘no choice’ leaves me feeling extremely anxious… I simply can’t make a decision.”	- Perceived disappearance of relevant positions - Sense of having no viable choice - Restricted option space	1a. The reality shock of objectively scarce options	Theme 1: Structural Constraints as Lived Experience
[Participant 6]: “Seeing all the bad news… my current strategy is simply not to look. The more I see, the more anxious I get… So I just put it off.”	- Information avoidance behavior - Negative emotion blockage - Procrastination in decision-making	1b. Anxiety-Induced Information Blocking	
[Participant 4]: “It feels like exams nowadays have zero margin for error… If I’m not absolutely certain, I won’t dare sign up… Anxiety makes me feel incapable… though really, it’s just fear of taking action.”	- Fear of making the wrong move - Avoidance of uncertain attempts - Inability to act without certainty	2a. Catastrophising the cost of trial and error	Theme 2: Zero-Tolerance-for-Error Cognition in Career Decision-Making
[Participant 9]: “I graduated from a second-tier university… holding no advantage… this sense of educational inferiority intensifies, making me convinced I simply cannot pass.”	- Perceived disadvantage linked to university background - Sense of reduced competitiveness - Self-doubt about employability	2b. Self-doubt associated with educational background	
[Participant 5]: “My parents forwarded me loads of irrelevant job postings. This ‘support’ only made me feel they didn’t understand me at all, adding to my burden… increasing my anxiety.”	- Information mismatch - Increased cognitive load - Feeling misunderstood	3a. Resource mismatch and cognitive overload	Theme 3: Conditionality of Parental Career Support under Anxiety
[Participant 11]: “They constantly say ‘returning home for exams is for your own good’… making me feel disobedient if I don’t comply. This pressure makes it difficult to choose a city based on my own convictions.”	- Guilt associated with non-compliance - Perceived pressure to follow parental advice - Reduced sense of decision autonomy	3b. Autonomy constraint and guilt-laden pressure	

Note: Quotations translated from original Chinese records. Pseudonyms used to protect participants’ privacy. The themes represent participants’ subjective interpretations of their career decision-making experiences and should not be interpreted as evidence of causal mechanisms.

**Table 6:** Regression Results for the Mediation Component of the Conditional Process Model.

	CDSE			CDD		
	*B*	*β*	*SE*	*B*	*β*	*SE*
Constant	4.570 ***	—	0.164	2.219 ***	—	0.270
CA	−0.346 ***	−0.275	0.060	0.549 ***	0.412	0.060
CDSE	—		—	−0.183 ***	−0.173	0.048
*R* ^2^	0.076			0.239		
*F*	33.258 ***			63.500 ***		

Note. *N* = 407. *B* = unstandardized coefficients; *β* = standardized coefficients. *SE* = Standard Error. Bootstrapping resamples = 5000. Proportion of mediated effect (P_M_) = 10.3%. *** *p* < 0.001.

**Table 7:** Regression Results for the Conditional Process Model.

Variables	CDSE		CDD	
	*B*	*t*	*B*	*t*
CA	−0.346 ***	−5.767	0.539 ***	8.976
CDSE	-		−0.143 **	−2.82
PCS	-		−0.719 ***	−4.151
CA × PCS	-		0.235 ***	3.79
*R* ^2^	0.076		0.273	
*F*	33.258 ***		37.704 ***	
Δ*R*^2^ (Interaction)	—		0.026	

Note. *N* = 407. B = unstandardized coefficients. Δ*R*^2^ represents the unique variance explained by the interaction term. All predictors were mean-centered prior to analysis. ** *p* < 0.01, *** *p* < 0.001.

**Table 8:** Simple Slope Analysis of the Conditional Association Between Career Anxiety and Career Decision-Making Difficulties at Different Levels of Parental Career Support.

PCS Levels	Slope (*B*)	*SE*	*t*	95% CI
Low	0.363	0.074	4.928 ***	[0.219, 0.508]
Medium	0.539	0.060	8.976 ***	[0.421, 0.657]
High	0.715	0.078	9.162 ***	[0.562, 0.868]

Note. CI = Confidence Interval. Low PCS = −1 *SD*; High PCS = +1 *SD*. *** *p* < 0.001.

## Data Availability

The data presented in this study are available on request from the corresponding author. The data are not publicly available due to privacy and ethical restrictions.

## Abbreviations

The following abbreviations are used in this manuscript:

SCCT	Social Cognitive Career Theory
CDD	Career Decision-Making Difficulties
CDSE	Career Decision-Making Self-Efficacy
PCS	Parental Career Support
CA	Career Anxiety
CFA	Confirmatory Factor Analysis
SEM	Structural Equation Modeling
CI	Confidence Interval
